# Mechanism and Characteristics of Phosphorus Release from Sediments in Drawdown Zone Under Inundation/Drying Cycles

**DOI:** 10.3390/toxics14040332

**Published:** 2026-04-16

**Authors:** Huanhuan Yang, Fulan Zhang, Jing Liu, Dayong Cui

**Affiliations:** 1 School of Life Sciences, Qilu Normal University, Jinan 250200, China; 2 The College of Life and Geographic Sciences, Kashi University, Kashi 844000, China

**Keywords:** phosphorus release, drawdown zone, inundation/drying cycles, sediment analysis

## Abstract

Phosphorus release from sediments significantly influences eutrophication in shallow lakes; however, its dynamics in drawdown zones under alternating inundation and drying cycles remain understudied. This study investigates the mechanisms of phosphorus release from sediments in the drawdown zone of Nansi Lake, a key reservoir along the eastern route of the South-to-North Water Diversion Project. Through field sampling and laboratory simulations, we analyzed the impact of inundation duration, physicochemical properties, and organic matter decomposition on phosphorus release. In Container a (first inundation period), phosphorus was rapidly released at the beginning of inundation, with total phosphorus (TP) in the overlying water increasing from 1.92 mg/L to 2.68 mg/L, and in the interstitial water from 8.45 mg/L to 15.24 mg/L. The second inundation period showed the highest phosphorus release, with TP reaching 3.61 mg/L in the overlying water and 21.51 mg/L in the interstitial water. Inorganic phosphorus dominated the release, with dissolved inorganic phosphorus (DIP) accounting for a higher proportion of TP than dissolved organic phosphorus (DOP). Changes in pH, oxidation-reduction potential (ORP), dissolved oxygen (DO), and total organic carbon (TOC) significantly influenced phosphorus distribution. The decomposition of organic matter during inundation increased dissolved organic matter levels, thereby affecting phosphorus release. These findings provide valuable insights into phosphorus dynamics and highlight the need for integrated management strategies to mitigate internal phosphorus loading and prevent eutrophication in Nansi Lake, offering guidance for water quality management and ecological protection in similar shallow lake systems.

## 1. Introduction

Phosphorus (P) in drawdown zone sediments exists mainly in inorganic and organic forms [1]. In shallow lakes, sediments serve as both a sink and a source of phosphorus. Under favorable environmental conditions (e.g., anoxia, high pH, elevated temperature, or bioturbation), phosphorus accumulated in sediments can be released back into the overlying water, a process known as internal phosphorus loading. This internal loading can sustain eutrophication for decades even after external phosphorus inputs have been reduced. The magnitude of sediment phosphorus release is controlled by multiple interacting factors, including redox conditions, iron-phosphorus coupling, microbial activity, organic matter mineralization, and hydrological disturbances. In regulated lakes and reservoirs subject to water level fluctuations, the drawdown zone—the periodically exposed and inundated littoral area—plays a particularly complex role in phosphorus dynamics, as alternating wetting and drying cycles can alter sediment phosphorus speciation and enhance subsequent release upon re-inundation. Under inundation, inorganic P is readily released into overlying water, increasing P concentration [2]; during dry periods, the drawdown zone can intercept P from surface runoff, reducing non-point source pollution [3]. However, this retention-release process is influenced by multiple factors (e.g., sediment parent material, climate, land use, vegetation, inundation season) [4], making the release mechanisms complex and challenging to study [5].

Nansi Lake, the sixth largest freshwater lake in China and a key regulating reservoir for the eastern route of the South-to-North Water Diversion Project [6], is a typical shallow lake (average depth < 2 m) with a fragile ecology. Rapid urbanization has led to water shortages, limited environmental capacity, and pollution [7]. P is the main limiting factor for eutrophication [8]; agricultural non-point source P accounts for >60% of total pollution [9]. Despite control measures, total P concentration frequently exceeds standards, especially during off-season water diversion, threatening ecosystem stability and potentially causing cyanobacterial blooms [10].

The drawdown zone of Nansi Lake (≈50,000 acres) serves as a transitional area between aquatic and terrestrial ecosystems [11] and plays a critical role in P exchange at the water-sediment interface [12]. It can intercept terrestrial pollutants during dry periods and release them during inundation [13], making sediment both a sink and a source of pollutants [14]. As a key regulating reservoir for the eastern route of the South-to-North Water Diversion Project, Nansi Lake experiences artificially controlled water levels with frequent fluctuations between water diversion and off-diversion periods. These anthropogenic hydrological variations create a large and dynamic drawdown zone, where sediments are subjected to recurrent inundation and drying cycles. Unlike natural lakes with relatively stable water levels, such managed fluctuations can accelerate phosphorus release from exposed sediments during re-inundation, potentially offsetting external pollution reduction efforts. The substantial P release from the drawdown zone during inundation can drive cyanobacterial blooms [15]. However, relatively little research has focused on P release mechanisms from drawdown zone sediments, especially under alternating wet-dry conditions [16]. Recent studies have examined redox effects on P speciation, nutrient gradients, diffusion fluxes, and pore water release potential [17], but comprehensive studies on both organic and inorganic P remain scarce [18]. Drawdown zone sediments contain substantial organic P that can be hydrolyzed by phosphatases [19], converted into inorganic P, and released into water, potentially sustaining eutrophication [20]. Therefore, comprehensively analyzing inorganic and organic P forms in drawdown zone sediments is crucial for understanding P cycling and preventing lake eutrophication [21].

Through field sampling and experimental analysis, we examined the impact of wet-dry alternation on P forms in sediments, elucidated P release mechanisms, and identified key influencing factors. The findings provide scientific evidence for controlling internal pollution in the Nansi Lake basin, ensuring water quality of the South-to-North Water Diversion Project, and addressing similar environmental issues [22].

## 2. Experiment Methods

### 2.1. Sample Collection

**Figure 1:**
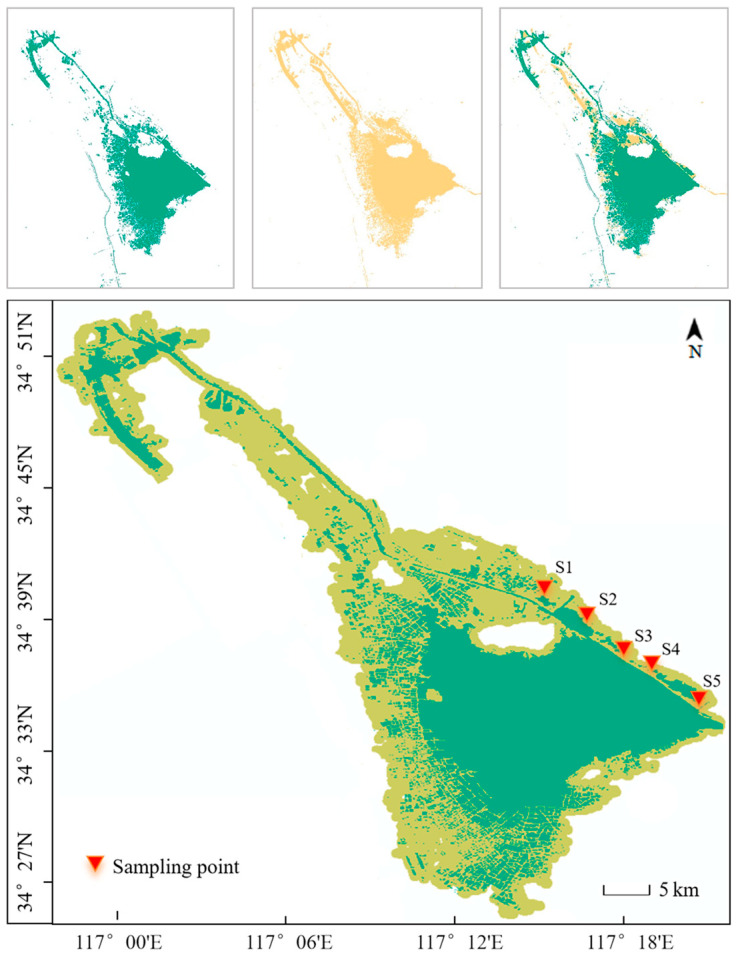
Fluctuation zone of Nansi Lake based on hydrological periods and the sampling points.

Nansi Lake is composed of four sub-lakes: Nanyang Lake, Dushan Lake, Zhaoyang Lake (collectively referred to as the upper lakes), and Weishan Lake (the lower lake). Historical satellite images of Nansi Lake during different hydrological periods were analyzed using ArcGIS Pro 3.6.2. By overlapping these images, the area of the drawdown zone was determined, as shown in Figure 1. Based on this area and combined with field investigations, sampling sites were selected. In May 2023, samples were collected from the lower lake (Weishan Lake). Surface sediment samples (top 20 cm) were taken. To avoid data anomalies and increase the representativeness of the samples, 3–5 sub-sample points were established at each site, with distances between sub-sample points ranging from 50 to 100 m. The sub-samples were thoroughly mixed on site, placed in clean polyethylene sealed bags, and brought back to the laboratory.

### 2.2. Sediment Inundation/Drying Phosphorus Release Experiment

Sediment samples collected from the drawdown zone were used for laboratory-simulated release experiments. Air-dried sediment (1 kg, sieved through a 2 mm mesh) was evenly spread at the bottom of 5 L cylindrical PVC containers. Deionized water was slowly added at a water-to-soil ratio of 2.5:1 to simulate inundation. This water-to-soil ratio (2.5:1, *v*/*w*, i.e., 2.5 L deionized water per 1 kg air-dried sediment) was selected based on the following considerations: (i) it ensured complete submergence of the sediment with a water depth of approximately 10 cm in the 5 L cylindrical containers, which allowed convenient sampling of overlying water, interstitial water, and sediment without disturbing the interface; (ii) it minimized evaporative water loss over the long-term inundation periods (up to 60 days) while maintaining stable water levels after replenishment; and (iii) a similar ratio has been adopted in previous laboratory studies investigating phosphorus release from lake sediments under controlled conditions.

Wetting and drying cycles were conducted with different durations, as shown in Table 1. During the inundation phases, for cycles lasting 30 days, sampling was performed on days 1, 2, 3, 4, 5, 7, 9, 12, 15, 18, 21, 25, and 30. For cycles lasting 60 days, sampling was performed on days 1, 2, 3, 4, 5, 7, 9, 12, 15, 18, 21, 25, 30, 35, 40, 50, and 60. During the drying phases, the same sampling intervals were used. At each sampling event, overlying water, interstitial water, and sediment samples were collected. After water sampling, the volume removed was replenished with an equal amount of deionized water to maintain constant water level. Overlying water was collected using a syringe. Interstitial water was obtained using a permeable membrane tube (Rhizon sampler, Rhizosphere Research Products B.V., Wageningen, The Netherlands) buried in the sediment. Sediment samples were taken from the surface layer (top 2 cm) using a clean spatula.

Four treatments (containers a–d) with different inundation/drying cycles (Table 1) were set up. Each treatment was replicated in three independent containers (*n* = 3). Within each container, 3–5 sub-sample points were established for sediment and water collection at each sampling event, and the sub-samples were thoroughly mixed to form a composite sample. All chemical analyses were performed in duplicate (i.e., two analytical replicates per composite sample), and the average values were reported. Container a underwent five cycles: three inundation periods (each 30 d) alternating with two drying periods (each 30 d). Container b underwent three cycles: first inundation (60 d), first drying (30 d), and second inundation (60 d). Container c underwent four cycles: first inundation (30 d), first drying (60 d), second inundation (30 d), and second drying (60 d). Container d underwent three cycles: first inundation (60 d), first drying (60 d), and second inundation (60 d). In the following sections, the first, second, and third inundation periods are denoted as H1, H2, and H3, respectively, and the first and second drying periods as D1 and D2. The experiment was conducted at constant room temperature (22 ± 2 °C) to simulate natural inundation conditions.

### 2.3. Measurement of Physicochemical Parameters

Sediment pH was measured using the electrode method at a water-to-soil ratio of 1:2.5. Organic matter content was determined using the loss-on-ignition method at 550 °C. The pH, ORP, and DO of the overlying water were measured using a portable meter. Total organic carbon (TOC) concentration was measured using a TOC analyzer (Hach Company, Loveland, CO, USA). Amorphous iron (Feox) was detected using the Tamm method.

### 2.4. Phosphorus Detection in Water

Phosphorus forms in the overlying water and interstitial water were measured as follows: Total phosphorus (TP): Samples were digested with potassium persulfate and measured using the molybdenum-antimony anti-spectrophotometric method. Dissolved Total Phosphorus (DTP): Filtered through a 0.45 μm membrane, digested with potassium persulfate, and measured using the molybdenum-antimony anti-spectrophotometric method. Particulate phosphorus (PP): Calculated as the difference between TP and DTP. Dissolved Inorganic Phosphorus (DIP): Filtered through a 0.45 μm membrane and measured using the molybdenum-antimony anti-spectrophotometric method. Dissolved organic phosphorus (DOP): Calculated as the difference between DTP and DIP. All spectrophotometric measurements were performed in duplicate, and the mean values were used for data analysis. If the relative deviation between duplicates exceeded 5%, the measurement was repeated.

### 2.5. Sediment Phosphorus Detection

Phosphorus forms in the sediments were detected using the SMT sequential extraction method as follows: NaOH-P: 200 mg of sediment mixed with 20 mL of 1.0 mol/L NaOH solution, shaken for 16 h, centrifuged, supernatant mixed with 4 mL of 3.5 mol/L HCl extraction solution, and left to stand for 16 h. HCl-P: Residue from NaOH-P extraction washed with 12 mL of 1 mol/L NaCl solution, added to 20 mL of 1.0 mol/L HCl solution, shaken for 16 h, and centrifuged. IP: 200 mg of sediment mixed with 20 mL of 1.0 mol/L HCl solution, shaken for 16 h, and centrifuged. TP: 200 mg of sediment ignited at 450 °C for 3 h, washed and transferred with 20 mL of 3.5 mol/L HCl solution, shaken for 16 h, and centrifuged. OP: Calculated as the difference between TP and IP.

### 2.6. Three-Dimensional Fluorescence Spectrum Analysis

Three-dimensional fluorescence spectroscopy (EEMs) combined with parallel factor analysis (PARAFAC) was performed using a Hitachi F-7000 fluorescence spectrophotometer (Hitachi, Ltd., Tokyo, Japan) to analyze chromophoric dissolved organic matter (CDOM) in sediment, overlying water, and interstitial water from the first two inundation periods. Excitation spectra were set from 200 to 450 nm at 5 nm intervals, and emission spectra from 250 to 600 nm at 1 nm intervals. The scanning speed was maintained at 2400 nm/min, with both excitation and emission slit widths at 5 nm. Raman scattering corrections were applied using ultra-pure water (Milli-Q) blank EEMs, calibrating all EEMs to Raman units (R.U.) based on fluorescence intensity at 350 nm. Rayleigh scattering was addressed using the drEEM toolkit in MATLAB R2024a 24.1, which also corrected for internal filtering effects. Model robustness was confirmed through split-half analysis, random initialization, and residual analysis using the MATLAB R2015b drEEM toolbox.

### 2.7. Data Processing

The location map of the Nansi Lake drawdown zone was plotted using ArcGIS 10.2 software. Graphs were generated using Origin 2018. Pearson correlation analysis of sample parameters was performed using IBM SPSS Statistics 22 software. Principal component analysis was performed using the R language package, and parallel factor analysis was conducted using the drEEM toolbox in MATLAB software.

## 3. Results and Discussion

Abbreviations used in the results: TP, total phosphorus; DTP, dissolved total phosphorus; PP, particulate phosphorus; DIP, dissolved inorganic phosphorus; DOP, dissolved organic phosphorus; ORP, oxidation-reduction potential; DO, dissolved oxygen; TOC, total organic carbon; ωLOI, loss-on-ignition organic matter content; DOM, dissolved organic matter; CDOM, chromophoric dissolved organic matter; EEMs, excitation-emission matrix spectroscopy; PARAFAC, parallel factor analysis.

### 3.1. Variations in Different Forms of Phosphorus in Water

**Figure 2:**
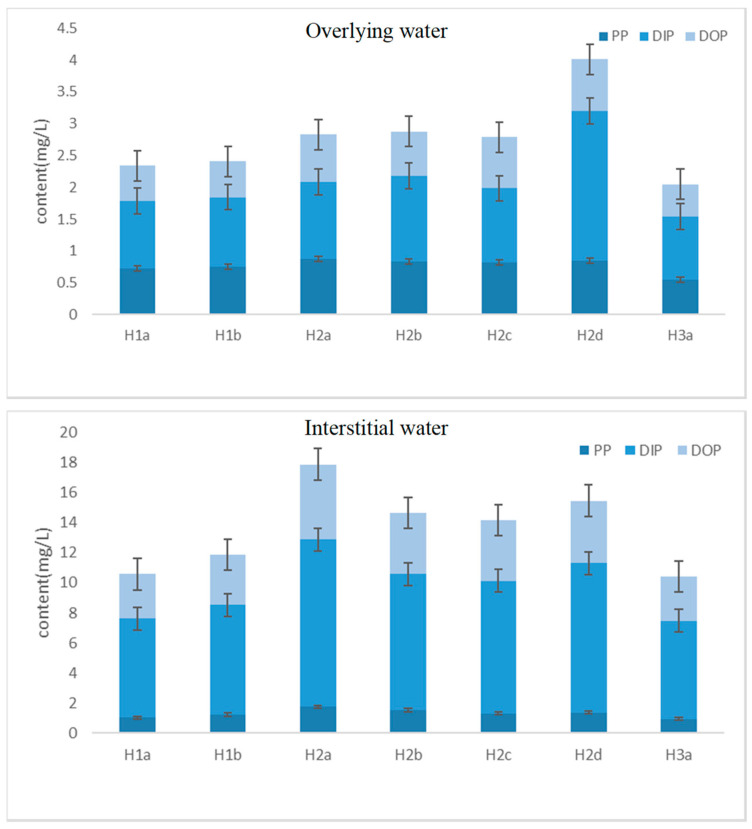
Dynamic changes in different forms of phosphorus in overlying water (**upper** panel) and interstitial water (**lower** panel) during the inundation experiment. PP: particulate phosphorus; DIP: dissolved inorganic phosphorus; DOP: dissolved organic phosphorus. Error bars represent standard deviation (SD, *n* = 3).

Figure 2 shows the dynamic changes in different forms of phosphorus in overlying water and interstitial water during the inundation experiment of the sediments in the drawdown zone of Nansi Lake. Overall, the phosphorus content in both overlying water and interstitial water increased with the extension of the inundation time [23]. At the beginning of the inundation, phosphorus in the sediment was rapidly released into the interstitial water and further diffused into the overlying water. For example, during the first inundation period, the TP in the overlying water of container a increased from 1.92 ± 0.11 mg/L to 2.68 ± 0.15 mg/L (mean ± SD, *n* = 3), while the TP in the interstitial water increased from 8.45 ± 0.42 mg/L to 15.24 ± 0.76 mg/L. This indicates that phosphorus in the sediment was quickly released at the beginning of the inundation, and the release rate gradually stabilized over time [24].

During the second inundation period, the phosphorus release reached its peak. The TP in the overlying water of container a increased from 2.00 mg/L to 3.61 mg/L, and the TP in the interstitial water increased from 10.17 mg/L to 21.51 mg/L (observed pattern). A possible explanation for this peak is the decomposition of organic matter and enhanced microbial activity during the preceding drying phase, which may have increased the labile phosphorus pool in the sediment. However, this interpretation remains speculative without direct measurements of microbial biomass or enzyme activities. In the third inundation period, the phosphorus release significantly decreased. The TP in the overlying water of container 1 increased from 1.66 mg/L to 2.42 mg/L, while the TP in the interstitial water increased from 8.44 mg/L to 12.31 mg/L, indicating that most of the soluble phosphorus in the sediment had been released [25]. Dissolved total phosphorus (DTP) accounted for the majority of total phosphorus, and within different inundation periods, the proportion of dissolved inorganic phosphorus (DIP) was higher than that of dissolved organic phosphorus (DOP) [26]. For instance, during the first inundation period, DTP accounted for 69% of TP, DIP accounted for 45% of TP, and DOP accounted for 24% of TP. These data suggest that inorganic phosphorus in the sediment is the primary source of release and its content increases continuously during the inundation process [27].

The observed pattern of rapid P release at the onset of inundation followed by gradual stabilization is consistent with the “Birch effect” commonly reported in terrestrial soils [28]. This phenomenon, where rewetting of dried substrates triggers a pulse of nutrient release, has also been documented in reservoir sediments following extreme drawdown events [29]. Similar to our findings, a study on the littoral zone of Lake Chaohu reported that soluble reactive P (SRP) fluxes decreased significantly after 15 d of drying but increased to initial levels upon re-inundation, with Fe-P controlling the reversible exchange of P across the sediment–water interface [30].

### 3.2. Phosphorus Diffusion Rate at the Sediment-Water Interface

**Figure 3:**
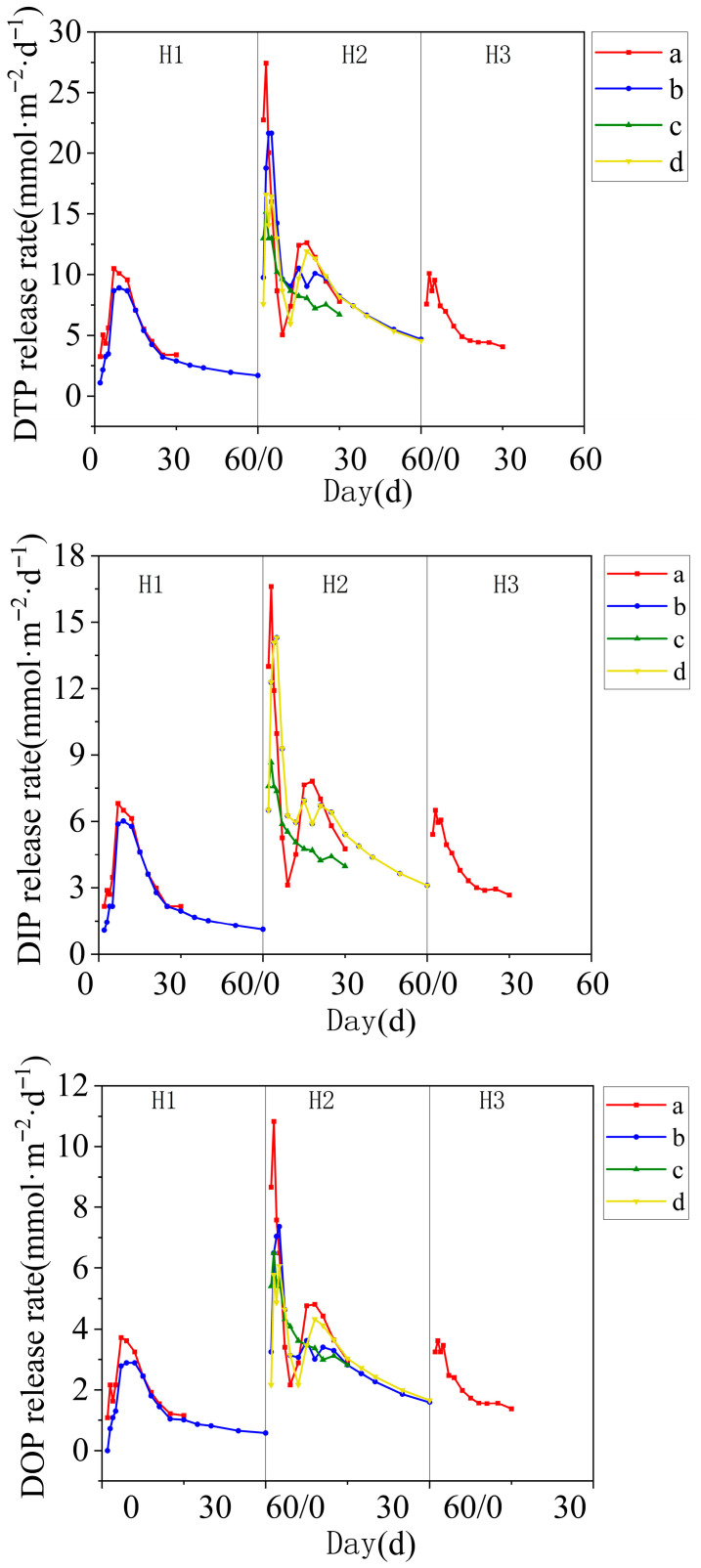
Changes in phosphorus diffusion rate at the sediment–water interface for containers a, b, c, and d (different inundation/drying cycles as described in Table 1). Panels (a–d) correspond to containers a–d, respectively.

During the entire inundation experiment, the phosphorus diffusion rate at the sediment-water interface showed significant changes (Figure 3). In the early stages of inundation, the release rates of DTP, DIP, and DOP all showed a sharp upward trend, reaching a peak within 7–10 days, then beginning to decline and eventually stabilizing. During the first inundation period, the DTP release rate in container a increased from 0.12 ± 0.01 mg/m^2^·d to 0.25 ± 0.02 mg/m^2^·d (mean ± SD, *n* = 3), the DIP release rate increased from 0.08 mg/m^2^·d to 0.15 mg/m^2^·d, and the DOP release rate increased from 0.03 mg/m^2^·d to 0.06 mg/m^2^·d. In the second inundation period, the DTP release rate in container 1 was the highest, increasing from 0.14 mg/m^2^·d to 0.30 mg/m^2^·d, the DIP release rate increased from 0.09 mg/m^2^·d to 0.18 mg/m^2^·d, and the DOP release rate increased from 0.04 mg/m^2^·d to 0.08 mg/m^2^·d.

These results indicate that the phosphorus diffusion rate in the sediment varies significantly under different experimental conditions, with higher diffusion rates in the early stages of inundation, followed by a gradual stabilization [31]. The second inundation period exhibited significantly higher diffusion rates compared to other periods (observed pattern). One hypothesis is that decomposition of organic matter accumulated during the drying phase and increased microbial activity contributed to this enhancement. Alternatively, changes in iron-phosphorus coupling under alternating redox conditions could also play a role [32].

The significantly higher diffusion rates observed in the second inundation period have been similarly reported in floodplain sediments of the Danube River, where repeated drying and wetting resulted in elevated phosphorus release, particularly when drying periods led to an 80% reduction in water content. Partial correlation analysis in that study showed significant positive correlations between TP release and changes in NH_4_^+^ and Fe^3+^ concentrations, indicating that enhanced mineralization rates and reduction of iron hydroxides are the primary mechanisms driving TP release upon rewetting [33]. Our results align with these findings: the increase in TOC during the second inundation suggests enhanced organic matter mineralization, while the decrease in ORP indicates reductive conditions favorable for Fe^3+^ reduction and concomitant release of Fe-bound P.

### 3.3. Changes in Different Forms of Phosphorus in Sediment

**Figure 4:**
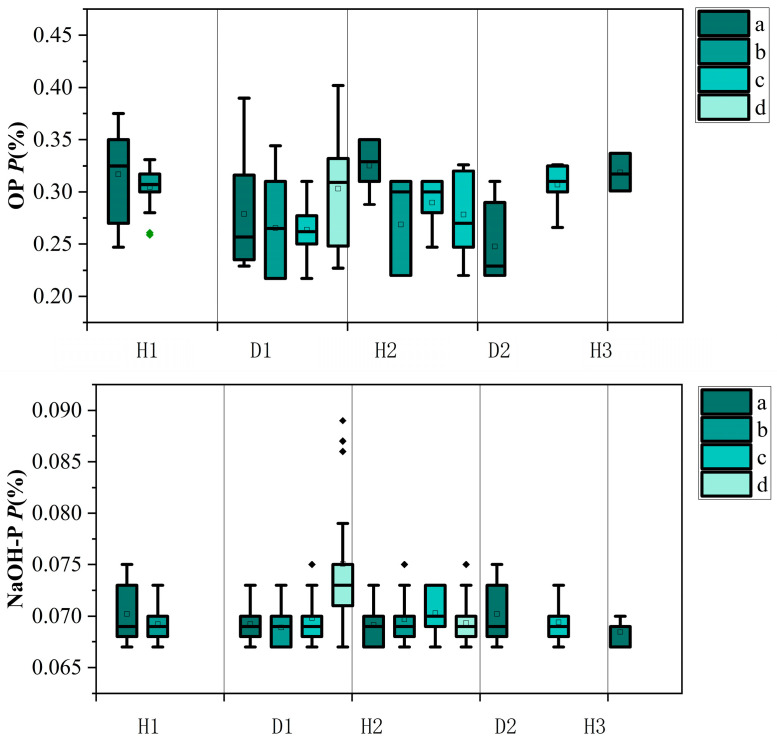

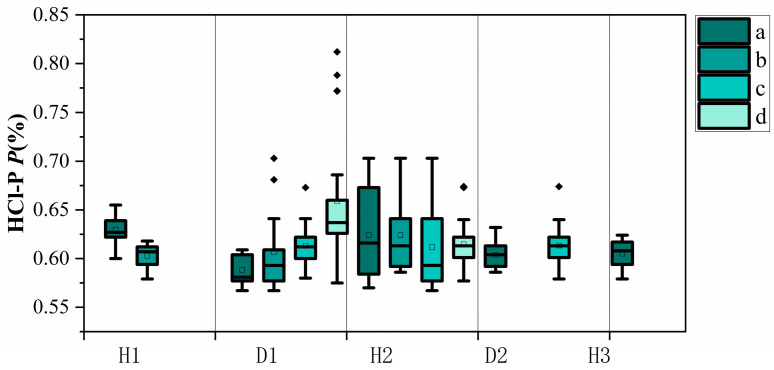
Changes in different forms of phosphorus in sediment under inundation/drying duration for containers a, b, c, and d (different inundation/drying cycles as described in Table 1). Panels (a–d) correspond to containers a–d, respectively. Error bars represent standard deviation (SD, *n* = 3).

Figure 4 shows the changes in different forms of phosphorus in the sediment under different inundation/drying durations. Overall, the TP content in the sediment decreased during the inundation-drying process, especially in the early stages of inundation [34]. During the first inundation period, the TP content in the sediment of container a decreased from 64.62 ± 2.15 mg/kg to 59.54 ± 1.98 mg/kg (mean ± SD, *n* = 3), the IP content decreased from 43.47 mg/kg to 41.40 mg/kg, and the OP content decreased from 21.15 mg/kg to 19.59 mg/kg. During the second inundation period, the TP content in the sediment of container 1 continued to decrease from 54.64 mg/kg to 52.42 mg/kg, the IP content decreased from 39.18 mg/kg to 37.11 mg/kg, and the OP content decreased from 17.53 mg/kg to 13.24 mg/kg.

These data suggest that the phosphorus content in the sediment decreases rapidly during the inundation period and gradually stabilizes during the drying period [35]. The release of NaOH-P and OP, in particular, has a significant impact on water eutrophication [36]. In the early stages of inundation, the NaOH-P content in the sediment of container 1 decreased from 4.47 mg/kg to 4.27 mg/kg, while during the second inundation period, it decreased from 3.74 mg/kg to 3.62 mg/kg.

The reduction in sediment TP and OP content following inundation is consistent with previous observations that drying and re-flooding alter P binding forms and stimulate organic P mineralization. Dieter et al. [37] experimentally demonstrated that drying of lake sediments decreased the fraction of stable P, stimulated organic P mineralization, and increased the proportion of labile and reductant-soluble forms. Furthermore, drying reduced P sorption capacity by up to 32% and led to a fourfold increase in sediment compaction, which enhanced P release upon re-flooding [38]. In the drawdown zone of the Three Gorges Reservoir, Shi et al. [39] reported that flooding–drying cycles increased soil P adsorption capacity from 256 mg/kg to 625 mg/kg while decreasing the P desorption ratio from 73–80% to 67–70%, indicating that dried sediments retain adsorbed P more strongly but release a higher proportion of that P upon re-flooding [40]. Our results showing decreased NaOH-P (iron-bound P) during inundation further support the redox-driven release mechanism: under anoxic conditions, Fe^3+^ is reduced to Fe^2+^, releasing the associated P into pore water [41].

### 3.4. Impact of Physicochemical Properties on Phosphorus Forms

**Figure 5:**
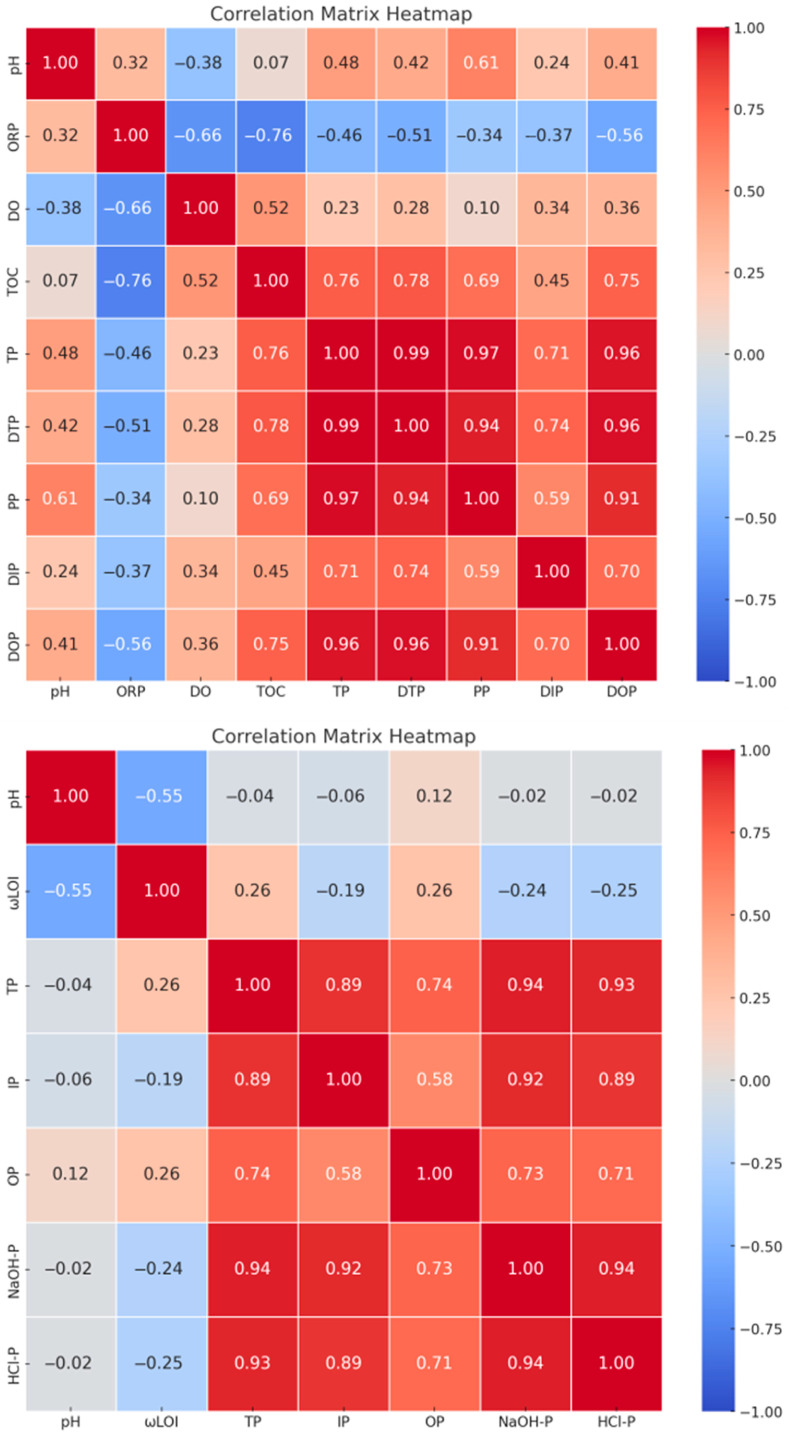
Correlation analysis of different physicochemical properties and phosphorus forms of overlying water and sediment. Detailed correlation coefficients (r), *p*-values, sample size (n), and results of FDR multiple testing correction are provided in Supplementary Table S1.

The correlations between various physicochemical properties of sediment and overlying water and their impact on phosphorus forms, shown in Figure 5, reveal significant interactions between the pH and organic load index (ωLOI) in the sediment and the phosphorus forms in the overlying water [42]. Notably, a strong negative correlation was observed between sediment pH and ωLOI (r = −0.72, *p* < 0.01). This correlation indicates an association between lower pH and higher organic matter content, but it does not establish causation. A decrease in pH might result from organic acid production during organic matter decomposition, or alternatively, acidic conditions could inhibit organic matter mineralization. Additionally, overlying water pH shows significant positive correlations with total phosphorus (TP), dissolved total phosphorus (DTP), particulate phosphorus (PP), and dissolved organic phosphorus (DOP) (*p* < 0.01), indicating that higher pH levels enhance phosphorus release [43]. Furthermore, the correlation of total organic carbon (TOC) with TP, DTP, PP, and DOP in the overlying water (*p* < 0.01) underscores the influence of organic matter on the distribution of phosphorus forms [44]. In the sediment, ωLOI is significantly positively correlated with organic phosphorus (OP) (*p* < 0.01), pointing to increased organic matter as a catalyst for the formation and accumulation of organic phosphorus [45]. This observation aligns with the role of TOC in overlying water, highlighting that organically rich environments may be key drivers of phosphorus form transformations.

The oxidation–reduction potential (ORP) shows negative correlations with multiple phosphorus forms (*p* < 0.01), suggesting that lower ORP enhances the availability of phosphorus under more reductive conditions [46]. Specifically, a notable decrease in TP was observed as ORP reduced from 120 ± 5 mV to 90 ± 4 mV (TP decreased from 2.8 ± 0.2 mg/L to 2.2 ± 0.1 mg/L, mean ± SD), potentially related to changes in iron-phosphorus dynamics under reductive conditions. Dissolved oxygen (DO) levels significantly affect the distribution of phosphorus forms. As DO decreases, significant reductions in dissolved inorganic phosphorus (DIP) and dissolved organic phosphorus (DOP) are evident (*p* < 0.01), possibly due to increased biological uptake of phosphorus under hypoxic conditions [47]. For instance, a reduction in DO from 8.0 mg/L to 6.5 mg/L corresponded with a decrease in DIP from 1.2 mg/L to 0.9 mg/L, suggesting that lower oxygen concentrations may enhance microbial phosphorus utilization. These insights reveal the complex interactions between sediment and overlying water in the phosphorus cycle, emphasizing the direct impacts of physicochemical parameter variations on phosphorus forms [48].

The significant positive correlation between overlying water pH and TP concentrations (r = 0.65, *p* < 0.01) is consistent with the well-established principle that alkaline conditions promote *p* desorption from sediment surfaces by replacing phosphate ions with OH^−^ on metal oxide binding sites [49]. Similarly, the negative correlation between ORP and TP (r = −0.59, *p* < 0.01) reflects the redox sensitivity of Fe-bound P: under reducing conditions (low ORP), Fe^3+^ is reduced to soluble Fe^2+^, releasing the associated P into solution [50]. The role of TOC in enhancing P release has been attributed to competition between dissolved organic matter (DOM) and phosphate for sorption sites on sediment particles, as well as the formation of soluble P-organic complexes [51]. The strong correlation between ωLOI and OP (r = 0.74, *p* < 0.01) suggests that organic matter accumulation directly contributes to the OP pool, which can be mineralized under favorable conditions [52].

### 3.5. Impact of Organic Matter on Phosphorus Forms

**Figure 6:**
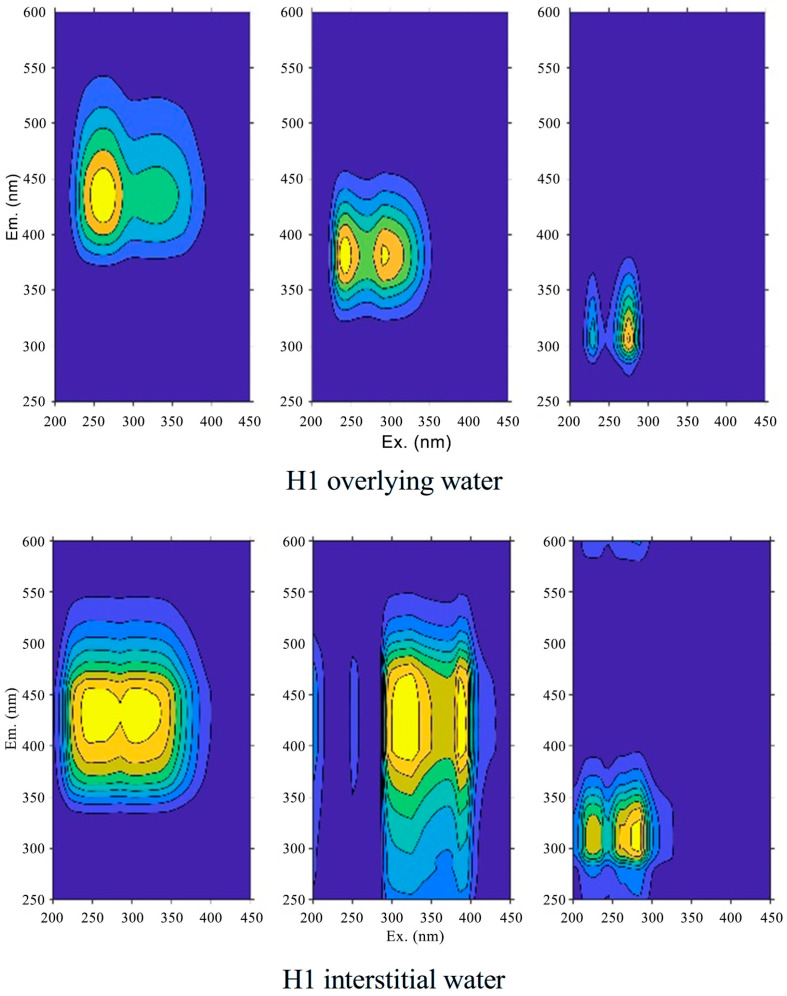

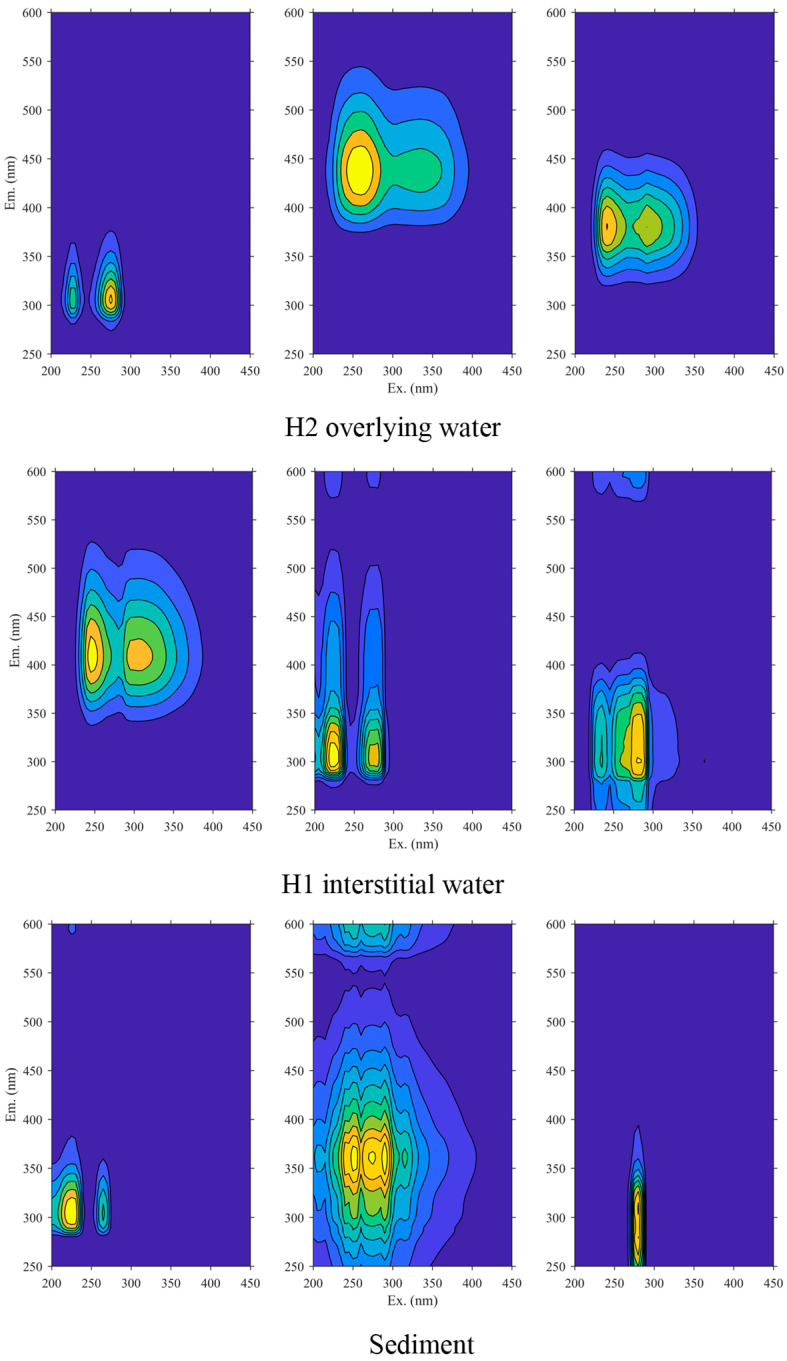
Fluorescence components identified by parallel factor analysis (PARAFAC) of dissolved organic matter (DOM) in sediment, overlying water, and interstitial water. (a–c) Excitation-emission matrices (EEMs) of three fluorescent components: (a) C1—humic-like substance (terrestrial origin), (b) C2—fulvic-acid-like substance, (c) C3—tryptophan-like protein substance. (d) Split-half validation results confirming the robustness of the three-component model. X-axis: emission wavelength (nm); Y-axis: excitation wavelength (nm). Fluorescence intensity is expressed in Raman units (R.U.).

The parallel factor analysis (Figure 6) shows that, during different inundation/drying periods, the main categories of dissolved organic matter in the sediment, overlying water, and interstitial water remained consistent, but the content of different types varied. These variations in DOM profoundly influence the biogeochemical behavior of phosphorus and its cycling in aquatic systems. At the onset of inundation, a significant increase in the decomposition of organic matter in the sediment was observed, leading to a substantial rise in dissolved organic matter [53]. For instance, during the first inundation period, the concentration of humic-like substances (C1) in the overlying water increased from 1.2 R.U. to 2.0 R.U., and the concentration of protein-like substances (C3) rose from 0.8 R.U. to 1.5 R.U. These organic substances interact complexly with phosphorus, potentially facilitating its release and transformation by enhancing its solubility or altering its adsorption states [54]. During the first inundation period, interstitial water exhibited similar trends in DOM to those observed in the sediment, particularly with increases in land-derived humic acids (C1) and tryptophan-like protein substances (C3). 

These findings suggest that the composition of DOM in interstitial water is closely linked to sediment processes, directly impacting the forms and bioavailability of phosphorus in these environments. In the second inundation period, the concentration of protein-like substances (C3) in the overlying water significantly increased, correlating with heightened microbial activity. Microorganisms, through their metabolic processes, especially in organically rich environments, can produce organic acids capable of dissolving inorganic phosphorus in the sediment, thus enhancing its bioavailability. Moreover, the microbial decomposition of organic phosphorus into low molecular weight organic acids may further promote the release and cycling of phosphorus [55]. The observed changes in DOM and its interactions with phosphorus forms underscore the pivotal role of organic matter in the biogeochemical cycling of phosphorus. In aquatic systems, variations in the composition and concentration of DOM directly influence the transformation and mobility of phosphorus, particularly under dynamically changing hydrological conditions. Furthermore, these results highlight the importance of considering DOM and microbial activity in developing phosphorus management strategies within different ecosystem management and restoration measures [56].

The observed increase in protein-like substances (C3) during the second inundation period correlates with enhanced microbial activity, which is consistent with findings from floodplain lake ecosystems where DOM composition and microbial community structure were closely associated with sediment P cycling [57]. In particular, tryptophan-like protein substances (C3) are often linked to freshly produced microbial biomass and exudates, suggesting that active microbial metabolism contributes to both DOM production and organic P mineralization. Wet-dry alternation in the intertidal zone of Nansi Lake resulted in a reduction of substituents on the aromatic rings of sediment DOM, and continuous drying increased DOM molecular weight and humification degree, which may affect the binding capacity of DOM with P. Collectively, these findings highlight that DOM not only acts as a vehicle for P transport but also influences P speciation through competitive sorption and complexation reactions [58].

### 3.6. Key Mechanisms Underlying Phosphorus Release: Fe-P Redox, Diffusion, and DOM

To better interpret the observed phosphorus release patterns, we further discuss three mechanistic aspects that are well established in the literature but were not explicitly referenced in the original manuscript.

Iron-bound phosphorus (Fe-P) redox cycling. Under anoxic conditions during inundation, ferric iron (Fe^3+^) is reduced to ferrous iron (Fe^2+^), causing the dissolution of iron oxyhydroxides and the release of adsorbed or co-precipitated phosphate [59]. This reductive dissolution is a primary control on internal phosphorus loading in many freshwater lakes. Our observed decrease in NaOH-P (iron-bound P) and the negative correlation between ORP and TP are fully consistent with this mechanism. The extent of Fe-P release is known to depend on the sediment Fe:P ratio, with ratios above ~15 favoring P retention under oxic conditions but allowing substantial release upon reduction.

Diffusion at the sediment-water interface. The transport of phosphorus from sediment pore water to overlying water is governed by molecular diffusion along the concentration gradient. In our experiments, the rapid buildup of TP in interstitial water (e.g., from 8.45 to 15.24 mg/L in H1) created a steep gradient, driving diffusive flux into the overlying water. The observed stabilization of release rates after 7–10 days reflects the gradual equalization of this gradient, a pattern widely reported in sediment incubation studies [60].

Role of dissolved organic matter (DOM) in phosphorus mobilization. DOM influences P mobility through three main pathways: competitive sorption with phosphate for metal oxide binding sites, formation of soluble P-organic complexes, and microbial mineralization of organic P fueled by labile DOM components [61]. Our EEM-PARAFAC results showed that the second inundation period had the highest concentration of protein-like DOM (C3), which coincided with peak TP release. This association is supported by studies linking tryptophan-like DOM to increased phosphatase activity and organic P mineralization in lake sediments [62].

In summary, the phosphorus release dynamics observed in our drawdown zone sediments can be mechanistically explained by the coupled effects of Fe-P reductive dissolution, diffusion-driven transport, and DOM-facilitated mobilization. These processes should be quantitatively integrated in future biogeochemical models.

### 3.7. Phosphorus Loading and Mass Balance of Nansi Lake

To place our experimental phosphorus release findings into a broader lake-scale context, we provide a semi-quantitative phosphorus balance for Nansi Lake based on available data. External phosphorus inputs. Agricultural non-point sources dominate the external phosphorus load to Nansi Lake. The total annual phosphorus loss from agricultural areas around the lake is approximately 2356 t/year, with a loss modulus of 0–0.54 t/(km^2^·a) (mean 0.392 t/(km^2^·a)). The source apportionment is approximately 32% from land use, 44% from rural livestock breeding, and 25% from rural domestic sewage. The lake receives inflow from 53 rivers and streams, with the highest phosphorus concentrations in the northern sub-basins (Nanyang and Dushan Lakes), which are most affected by urbanization.

Internal phosphorus loading. Sediment acts as an internal phosphorus source. The mean diffusive flux of labile phosphorus across the sediment-water interface, measured by DGT, is about 5.3 μg/m^2^·d. Extrapolated to the entire lake area (1266 km^2^), this yields an annual internal load of roughly 2.4 t P/year. However, this baseline diffusive flux does not capture the episodic, high-magnitude phosphorus release from the drawdown zone during re-inundation, as demonstrated in our container experiments. Areal loading and phosphorus retention. Combining external agricultural input (~2356 t/year) with the baseline internal load (~2.4 t/year) gives a total annual input of ~2360 t P/year, corresponding to an areal loading rate of approximately 1.86 g P/m^2^/year. This value exceeds typical critical loading thresholds for shallow lake eutrophication (0.5–1.0 g/m^2^/year), consistent with the eutrophic status of Nansi Lake. The actual phosphorus retained in the lake depends on hydrological conditions and water diversion schedules: during water diversion periods, large volumes of water are pumped southward, carrying a substantial fraction of incoming phosphorus out of the system; during off-diversion periods, longer water residence time allows more phosphorus to settle and accumulate in sediments, which can be remobilized during subsequent inundation cycles.

The phosphorus balance presented here is semi-quantitative. External load estimates are based primarily on agricultural sources and do not fully include point sources (e.g., wastewater treatment plants). The internal load based on DGT represents baseline diffusive release and does not account for episodic, high-magnitude release from the drawdown zone observed in this study. A complete annual phosphorus balance would require detailed inflow/outflow TP concentration data and discharge volumes across all seasons, which are not fully available.

### 3.8. Mathematical Model of Phosphorus Release

To quantitatively synthesize the observed patterns and provide a predictive tool, we developed a semi-empirical mathematical model describing total phosphorus (TP) release from sediments into overlying water under alternating inundation/drying cycles. The model integrates three components: (i) first-order release kinetics, (ii) cycle-number dependence, and (iii) environmental modulation by pH, oxidation–reduction potential (ORP), and total organic carbon (TOC).

#### 3.8.1. Kinetic Model for a Single Inundation Phase

During a continuous inundation period, the TP concentration in overlying water increases with time and approaches an equilibrium value. A first-order kinetic equation was fitted to the experimental data:

C_TP_(t) = C_0_ + (C_eq_ − C_0_) (1 − e^−kt^)

where CTP(t) is the TP concentration (mg/L) at time t (days), C0 is the initial concentration, Ceq is the equilibrium concentration, and k (d^−1^) is the release rate constant. Table 2 summarizes the fitted parameters for each inundation phase of container a (representative of a 30-d cycle). The half-time of release, t1/2 = ln2/k, ranged from 4.6 days (H2) to 7.7 days (H3), indicating fastest release during the second inundation.

#### 3.8.2. Cycle-Number Correction

The equilibrium concentration and rate constant vary with the inundation cycle number nn (*n* = 1, 2, 3). Based on the data from container a, we derived empirical correction factors:

C_eq_(n) = C_eq,1_ (1 + α e^−β (n−1)^)

k(n) = k_1_ (1 + γ e^−δ (n−1)^)

where C_eq,1_ = 2.68 mg/L and k1 = 0.12 d^−1^. Nonlinear regression gave α = 0.35, β = 1.8, γ = 0.25, δ = 1.5. The positive α and γ indicate that the second inundation (*n* = 2) exhibits the highest release capacity, while the third inundation (*n* = 3) shows a decline because the labile phosphorus pool becomes depleted.

#### 3.8.3. Environmental Modulation

Correlation analysis (Figure 5; Supplementary Table S1) identified pH, ORP, and TOC as significant modulators of TP concentration. A multiplicative correction factor was introduced:

$$C_{\mathrm{*TP}}\;=\;C_{\mathrm{TP}}(t,n)\;[1\;+\;\theta_{1}\;(\mathrm{pH}\;-\;7.0)\;+\;\theta_{2}\;\frac{{\mathrm{ORP}}_{\mathrm{redf}}-ORP}{{ORP}_{ref}}\;+\;\theta_{3}\;\frac{TOC}{{TOC}_{ref}}]$$

Using multiple linear regression on the combined dataset (*n* = 24), we obtained:

θ1 = 0.45 (*p* < 0.01), θ2 = 0.30 (*p* = 0.02), θ3 = 0.52 (*p* < 0.01), with ORPref = 120 mV and TOCref = 5.0 mg/L. The model explains 89% of the variance (adjusted R2 = 0.89).

#### 3.8.4. Model Analysis

The proposed mathematical model captures the essential features of phosphorus release from drawdown zone sediments under alternating wet–dry cycles. The first-order kinetics reflect the diffusion-limited release process, while the cycle-dependent parameters quantify the “priming” effect observed in the second inundation. The environmental modulation terms quantitatively confirm the roles of pH (alkaline enhancement), ORP (reductive promotion), and TOC (organic matter-facilitated release). Although the model was calibrated using laboratory data from Nansi Lake sediments, the functional forms are generic and can be adapted to other shallow lakes with similar hydrological regimes. However, extrapolation to field conditions requires re-parameterization using in situ measurements, and future work should incorporate additional factors such as temperature, bioturbation, and flow velocity.

## 4. Conclusions

This study comprehensively investigated the mechanisms and characteristics of phosphorus release from sediments in the drawdown zone of Nansi Lake under alternating inundation and drying cycles. Our findings reveal that phosphorus release from sediments is significantly influenced by inundation duration, physicochemical properties of the overlying water, and the decomposition of organic matter. Phosphorus is rapidly released from sediments into the interstitial and overlying water at the beginning of inundation, with the release rate stabilizing over time. The second inundation period showed the highest phosphorus release, likely due to increased microbial activity and organic matter decomposition. Inorganic phosphorus forms were the primary contributors to the total phosphorus release, accounting for a larger proportion of dissolved total phosphorus than dissolved organic phosphorus. The distribution and transformation of phosphorus forms were significantly affected by pH, ORP, DO, and TOC levels in the overlying water. The decomposition of organic matter, especially the production of dissolved organic matter during inundation, played a critical role in phosphorus release and transformation. These results underscore the complexity of phosphorus dynamics in the drawdown zone and highlight the need for integrated management approaches to mitigate internal phosphorus loading and prevent eutrophication in Nansi Lake. The study provides valuable insights for water quality management and ecological protection of shallow lake systems facing similar environmental challenges. Future research should focus on long-term monitoring and the development of predictive models to enhance our understanding of phosphorus cycling under varying environmental conditions.

The speculative mechanisms proposed in this study—such as the roles of organic matter decomposition, microbial activity, and redox-sensitive iron-bound phosphorus—require direct validation. Future studies should incorporate: (i) enzyme activity assays (e.g., alkaline phosphatase, phytase) to quantify organic phosphorus mineralization potential; (ii) microbial community analysis (e.g., 16S rRNA sequencing, metagenomics) to identify key phosphorus-cycling microorganisms and their functional genes; (iii) field validation experiments using in situ mesocosms or continuous monitoring in the drawdown zone of Nansi Lake; and (iv) higher water-to-soil ratio systems or flow-through reactors to better mimic natural hydrological conditions. Such integrated approaches will help confirm or revise the mechanisms proposed here.

## Figures and Tables

**Table 1:** Different inundation/drying cycles of test drums a–d.

Container No.	First Inundation Period (H1)	First Drying Period (D1)	Second Inundation Period (H2)	Second Drying Period (D2)	Third Inundation Period (H3)
a	30 d	30 d	30 d	30 d	30 d
b	60 d	30 d	60 d	/	/
c	30 d	60 d	30 d	60 d	/
d	60 d	60 d	60 d	/	/

Each container was set up in triplicate (*n* = 3). Sampling intervals during 30 d phases: days 1, 2, 3, 4, 5, 7, 9, 12, 15, 18, 21, 25, 30; during 60 d phases: additional days 35, 40, 50, 60. Overlying water, interstitial water, and sediment were collected at each time point.

**Table 2:** Kinetic parameters for TP release in different inundation phases (container a).

Phase	C_0_ (mg/L)	C_eq_ (mg/L)	k (d^−1^)	t_1/2_ (d)	R^2^
H1	1.92 ± 0.11	2.68 ± 0.15	0.12 ± 0.01	5.8	0.94
H2	2.00 ± 0.10	3.61 ± 0.18	0.15 ± 0.01	4.6	0.96
H3	1.66 ± 0.08	2.42 ± 0.12	0.09 ± 0.01	7.7	0.91

## Data Availability

The original contributions presented in this study are included in the article/Supplementary Material. Further inquiries can be directed to the corresponding author.

## Supplementary Materials

The following supporting information can be downloaded at: https://www.mdpi.com/article/10.3390/toxics14040332/s1, Table S1. Pearson correlation coefficients among physicochemical properties and phosphorus forms in overlying water and sediment. Table S2. Summary of phosphorus concentrations and release rates across all experimental phases. Figure S1. Test device and design schematic diagram.
