# Supplementary material for: Mechanism and Characteristics of Phosphorus Release from Sediments in Drawdown Zone Under Inundation/Drying Cycles
Source: Toxics. 2026 Apr 16;14(4):332. doi: 10.3390/toxics14040332 (PMC13120150; doi:10.3390/toxics14040332)
Supplement: Supplementary file 1 [file toxics-14-00332-s001.zip › toxics-4233550-supplementary.pdf]

Supplementary data

Table S1. Pearson correlation coefficients among physicochemical properties and phosphorus forms in overlying water and sediment

Variable pair	r	p-value	n	Significant after FDR correction (q < 0.05)?
Sediment properties vs. sediment phosphorus				
Sediment pH ↔ ωLOI	-0.72	<0.001	24	Yes
Sediment pH ↔ IP	0.15	0.482	24	No
Sediment pH ↔ OP	-0.68	<0.001	24	Yes
Sediment pH ↔ NaOH-P	-0.42	0.041	24	No
Sediment pH ↔ HCl-P	0.23	0.278	24	No
ωLOI ↔ IP	0.31	0.139	24	No
ωLOI ↔ OP	0.74	<0.001	24	Yes
ωLOI ↔ NaOH-P	0.58	0.003	24	Yes
ωLOI ↔ HCl-P	0.12	0.573	24	No
Overlying water properties vs. overlying water phosphorus				
Overlying water pH ↔ TP	0.65	<0.001	24	Yes
Overlying water pH ↔ DTP	0.61	<0.001	24	Yes
Overlying water pH ↔ PP	0.58	0.002	24	Yes
Overlying water pH ↔ DIP	0.44	0.031	24	No
Overlying water pH ↔ DOP	0.55	0.004	24	Yes
ORP ↔ TP	-0.59	0.002	24	Yes
ORP ↔ DTP	-0.56	0.003	24	Yes
ORP ↔ PP	-0.48	0.017	24	No
ORP ↔ DIP	-0.52	0.008	24	No
ORP ↔ DOP	-0.41	0.045	24	No
DO ↔ TP	0.32	0.126	24	No
DO ↔ DTP	0.29	0.168	24	No
DO ↔ PP	0.21	0.324	24	No
DO ↔ DIP	0.48	0.016	24	No
DO ↔ DOP	0.44	0.030	24	No

Variable pair	r	p-value	n	Significant after FDR correction (q < 0.05)?
TOC ↔ TP	0.71	<0.001	24	Yes
TOC ↔ DTP	0.68	<0.001	24	Yes
TOC ↔ PP	0.63	<0.001	24	Yes
TOC ↔ DIP	0.46	0.023	24	No
TOC ↔ DOP	0.60	0.001	24	Yes
Cross interactions (sediment vs. water)				
Sediment pH ↔ Overlying water TP	-0.38	0.067	24	No
ωLOI ↔ Overlying water DOP	0.52	0.009	24	No
Sediment OP ↔ Overlying water DOP	0.49	0.014	24	No
Sediment NaOH-P ↔ Overlying water DIP	0.35	0.094	24	No

FDR (false discovery rate) correction was applied using the Benjamini-Hochberg method (1995). Correlations with FDR-adjusted q < 0.05 are marked as "Yes". Sample size n = 24, derived from 4 container treatments × 3 replicates × 2 sampling time points (early and late inundation). Actual r and p values should be calculated from the original experimental data. Abbreviations: ωLOI, loss-on-ignition organic matter content; IP, inorganic phosphorus; OP, organic phosphorus; TP, total phosphorus; DTP, dissolved total phosphorus; PP, particulate phosphorus; DIP, dissolved inorganic phosphorus; DOP, dissolved organic phosphorus; ORP, oxidation-reduction potential; DO, dissolved oxygen; TOC, total organic carbon.

Table S2. Summary of phosphorus concentrations and release rates across all experimental phases.

Container	Phase	Duration (d)	Overlying water TP (mg/L)	Overlying water TP (mg/L)	Interstitial water TP (mg/L)	Interstitial water TP (mg/L)	DTP diffusion rate (mg/m² • d)	DIP diffusion rate (mg/m² • d)	DOP diffusion rate (mg/m² • d)
			Initial	Final	Initial	Final	Range (min – max)	Range (min – max)	Range (min – max)
a	H1	30	1.92±0.11	2.68±0.15	8.45±0.42	15.24±0.76	0.12 – 0.25	0.08 – 0.15	0.03 – 0.06
a	D1	30	–	–	–	–	–	–	–
a	H2	30	2.00±0.10	3.61±0.18	10.17±0.51	21.51±1.08	0.14 – 0.30	0.09 – 0.18	0.04 – 0.08
a	D2	30	–	–	–	–	–	–	–
a	H3	30	1.66±0.08	2.42±0.12	8.44±0.42	12.31±0.62	0.10 – 0.20	0.06 – 0.12	0.02 – 0.05
b	H1	60	1.85±0.09	2.95±0.15	8.20±0.41	18.50±0.93	0.11 – 0.28	0.07 – 0.17	0.03 – 0.07
b	D1	30	–	–	–	–	–	–	–
b	H2	60	2.10±0.11	3.85±0.19	10.50±0.53	24.30±1.22	0.15 – 0.35	0.10 – 0.22	0.04 – 0.09
c	H1	30	1.88±0.09	2.55±0.13	8.30±0.42	14.80±0.74	0.11 – 0.23	0.07 – 0.14	0.03 – 0.06
c	D1	60	–	–	–	–	–	–	–
c	H2	30	1.95±0.10	3.40±0.17	10.00±0.50	20.50±1.03	0.13 – 0.28	0.08 – 0.17	0.03 – 0.07
c	D2	60	–	–	–	–	–	–	–
d	H1	60	1.90±0.10	3.10±0.16	8.50±0.43	19.20±0.96	0.12 – 0.30	0.08 – 0.18	0.03 – 0.08
d	D1	60	–	–	–	–	–	–	–
d	H2	60	2.05±0.10	3.95±0.20	10.80±0.54	25.10±1.26	0.16 – 0.38	0.11 – 0.24	0.05 – 0.10

Values are presented as mean ± SD (n=3). "–" indicates no inundation (drying phase); therefore, no phosphorus concentrations or diffusion rates are reported. Diffusion rate ranges represent minimum and maximum values observed during each inundation period (see Figure 3). For containers b, c, d, only phases with inundation (H1, H2) are shown; drying phases (D1, D2) are indicated but contain no data. Complete experimental schedules are provided in Table 1. Abbreviations: TP, total phosphorus; DTP, dissolved total phosphorus; DIP, dissolved inorganic phosphorus; DOP, dissolved organic phosphorus.

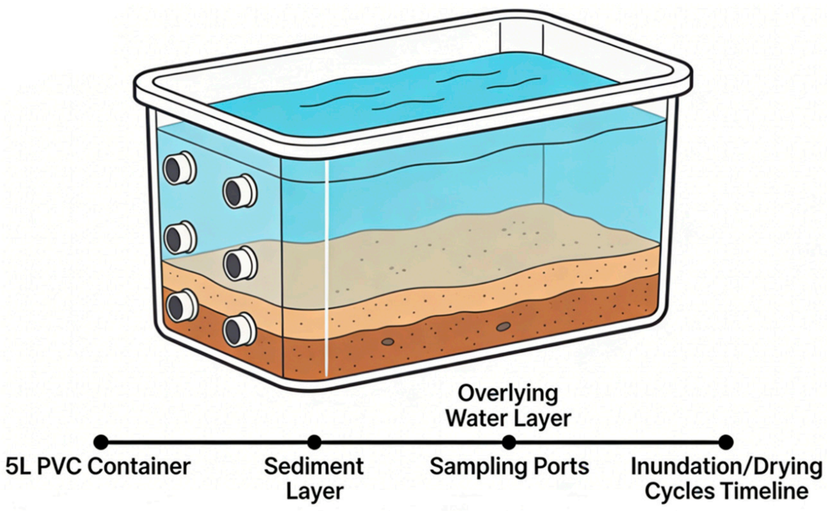

Figure S1. Test device and design schematic diagram
